# Association of Serum Pepsinogens With Esophageal Squamous Cell Carcinoma Risk: A Systematic Review and Meta-Analysis

**DOI:** 10.3389/fonc.2022.928672

**Published:** 2022-06-30

**Authors:** Zhen-Xiao Yang, Lu-Bin Yan, Peng Xie, Peng Hu, Wenjing Zhao, Yi Lu, Xiangbing Xing, Xudong Liu

**Affiliations:** ^1^ Department of Epidemiology, School of Public Health, Sun Yat-sen University, Guangzhou, China; ^2^ School of Public Health, Guangdong Pharmaceutical University, Guangzhou, China; ^3^ Department of Pediatric Surgery, The Sixth Affiliated Hospital, Sun Yat-sen University, Guangzhou, China; ^4^ Department of General Surgery, Xi’an Aerospace General Hospital, Xi’an, China; ^5^ School of Public Health and Emergency Management, Southern University of Science and Technology, Shenzhen, China; ^6^ Health Effects Institute, Boston, MA, United States; ^7^ Department of Gastroenterology, The First Affiliated Hospital of Sun Yat-sen University, Guangzhou, China

**Keywords:** serum pepsinogens, esophageal squamous cell carcinoma, systematic review, meta-analysis research, etiology

## Abstract

**Background:**

Serum pepsinogens are serological biomarkers of gastric atrophy, and the latter is a risk factor for esophageal squamous cell carcinoma (ESCC). However, the association of serum pepsinogens with ESCC risk remains unclear. This systematic review and meta-analysis aimed to assess the relationship between serum pepsinogen I (PGI) and pepsinogen I: pepsinogen II ratio (PGR) and ESCC risk.

**Methods:**

PubMed, Embase, and Web of Science were searched for articles on the effect of serum PGI and PGR on ESCC risk, published up to the end of February 2022. Meta-analysis with a random-effect model was used to calculate pooled odds ratios (ORs) and 95% confidence intervals (CIs).

**Results:**

Five case–control studies and three prospective studies were included. In comparison with the high categories, the low categories of serum PGI (OR: 1.92, 95% CI: 1.45–2.56) and PGR (OR: 1.70, 95% CI: 1.01–2.85) were associated with an increased risk of ESCC, although a substantial heterogeneity was observed in serum PGR (*I* ^2^ = 60.2%, *P* = 0.028) rather than in serum PGI (*I* ^2^ = 46.4%, *P* = 0.070). In stratified analysis by study quality, the significant risk effect on ESCC was remained for PGI (OR: 2.05, 95% CI: 1.48–2.84) and PGR (OR: 2.07, 95% CI: 1.17–3.75) when only the studies with high quality were pooled.

**Conclusions:**

Based on the available studies, although limited in number, this systematic review along with meta-analysis suggests that low serum PGI and low PGR may be related to an increased risk of ESCC. This present study provides evidence for using serum pepsinogen biomarkers in predicting ESCC. More delicate well-designed cohort studies with high study quality are needed, and dose–response analysis should be performed.

## Introduction

Esophageal cancer is one of the most common malignant tumors worldwide. It ranks seventh in cancer incidence and sixth in cancer mortality worldwide (1, 2). Esophageal squamous cell carcinoma (ESCC) is one of dominant histological subtypes, accounting for nearly 90% of all esophageal cancer cases in the world (3, 4). More than half of ESCC patients have progressed to the advanced stage when clinically diagnosed, and the overall 5-year survival rate is less than 20% (5, 6).

Early detection, early diagnosis, and early treatment can improve the patient’s condition and thereafter increase the survival of patients. Invasive and costly endoscopy and biopsy for histopathology are the gold standard for diagnosis of esophageal cancer (7). However, in most high-risk populations, the high cost and invasiveness of surgery are not acceptable. Therefore, it is necessary to develop simple, inexpensive, and accurate minimally invasive tools to identify asymptomatic high-risk individuals at the early stage. In recent years, many epidemiological studies have proved that blood biomarkers represent the most likely candidate indicators to promote the early detection of esophageal cancer (8–10). Gastric atrophy was evidenced to be a risk factor for ESCC (11, 12), and serum pepsinogens are serological biomarkers of gastric atrophy (13, 14). Serum pepsinogens are categorized into two groups: pepsinogen I (PGI) and pepsinogen II (PGII). The former is secreted by epithelium of the gastric body, and the latter is secreted by epithelium in both gastric antrum and gastric mucosa (15, 16). Atrophy of the gastric mucosa leads to a decrease in PGI, but no change in PGII, which further leads to a decrease in the pepsinogen I: pepsinogen II ratio (PGR). Several epidemiological studies found that low serum PGI and low serum PGR were associated with an increased risk of ESCC (17–21); however, other studies did not find such association (22–24).

Two previous meta-analyses (25, 26) examined the association by pooling potential studies, but the flaws in both studies affected the credibility and application of the results. Islami and colleagues (25) did the meta-analysis by synthesizing seven studies; nonetheless, two studies (27, 28) included in this meta-analysis did not examine the association between serum pepsinogens and risk of ESCC. The study by Yokoyama and colleagues examined the association of atrophic gastritis with risk of gastric cancer among Japanese ESCC male patients with alcoholic habits (27), and the study by Kamangar et al. examined the association of PGR with the risk of esophageal squamous dysplasia (28). Another meta-analysis (26) examined the association of ESCC risk with PGI and PGR by only pooling results from three studies (20, 22, 23); however, other epidemiological studies also examined the effect of PGI (17–19, 21, 24) and PGR (18, 19, 24) on ESCC risk; the incomplete inclusion of literature might lead to bias in the systematic review and meta-analysis.

Therefore, this systematic review and meta-analysis aimed to assess the association of ESCC risk with serum PGI and PGR by pooling the existing evidence.

## Methods

### Search Strategy and Selection Criteria

This systematic review and meta-analysis was conducted by following the PRISMA statement (29). The two researchers (Z-XY, WZ) independently searched the publications, reviewed the included studies, and extracted the relevant information from each study. Any disagreement between the third researchers was resolved by discussing with the third researchers (XL).

A systematic literature search for studies published in PubMed, Embase, and Web of Science was conducted up to the end of February 2022 for original studies in humans on the association between serum pepsinogens and ESCC, with no language restriction. The following search strategy was adopted: (“pepsinogen I” OR “pepsinogen II” OR “PGI” OR “PGII” OR “pepsinogen I: pepsinogen II ratio” OR “PGI: PGII” OR “PGI/II” OR “PGR” OR “PG” OR “pepsinogen” OR “gastric atrophy” OR “pepsinogens” [Mesh] OR ‘‘gastritis, atrophic’’[Mesh]) AND (“esophageal cancer” OR “esophageal carcinoma” OR “esophageal tumor” OR “esophageal neoplasms” OR “esophageal squamous dysplasia” OR “esophageal squamous cell carcinoma” OR “esophageal squamous cell carcinoma” [Mesh]). Reference and citation tracking was also conducted to retrieve potential studies. A more detailed search strategy is shown in **Supplementary Table S1**.

Eligible studies met the following criteria: study design of the case–control study or cohort study, ESCC cases were diagnosed by histological examination, the association of serum pepsinogens with ESCC incidence was examined, and the relative risk (RR) or odds ratios (ORs) and their 95% confidence interval (CIs) were reported or could be calculated. Cross-sectional studies, comments, editorials, animal studies, and abstracts were excluded. For multiple reports of the same biomarker from the same research population, only the most recent or informative report was included.

### Quality Assessment

The quality of included studies was evaluated using the Newcastle–Ottawa Scale (NOS) (30, 31). This scale contains eight items, which are categorized into three domains: selection of participants, comparability between participants in different subgroups, and assessment of exposure (case–control study) or outcome (cohort study). The total score ranged from 0 to 9, with a higher score indicating a better methodological quality. A study was assessed as low, moderate, or high quality if the total score was in the range of 0–3, 4–6, or 7–9 score, respectively.

### Data Extraction

The following information was collected from each included study, including first author, year of publication, country where the study was performed, study design, follow-up period for cohort studies, period when subjects were recruited for case–control studies, number of cases and total individuals for cohort studies, number of cases and controls for case–control studies, median or mean of age, male proportion, serum pepsinogen biomarkers and boundary point, risk estimates and their 95% CIs, and the covariates. The cutoff points for serum PGI and PGR varied in different studies. When the results for several cutoff points were provided in a study, we selected those which were more consistent with the cutoff points in other studies, that is, PGI <25 to ≤ 70 μg/l and PGR≤ 2 to ≤3.

### Statistical Analysis

A meta-analysis was conducted to evaluate the pooled effect of low level of serum PGI and PGR on the ESCC risk by comparing with the correspondingly high level. The random-effect model was used if the heterogeneity was observed (*I* ^2^ ≥ 50% and *P* ≤ 0.1); otherwise, the fixed-effect model was adopted (32). In order to facilitate understanding, OR was used to display the pooled effect. The heterogeneity among the studies was assessed using the *I* ^2^ statistic and the Q-test. When studies reported both crude and adjusted ORs and 95% CIs, the fully adjusted estimates were selected. Publication bias was assessed by funnel plot and Egger’s test (33, 34).

To explore potential sources of heterogeneity, several subgroup analyses were conducted. Since *Helicobacter pylori* was an important factor for ESCC, and the infection rate of *Helicobacter pylori* may be different in Western and Eastern countries (35), we performed a subgroup analysis based on the study region (Asia or Europe). Compared with the case–control study, the cohort study is more capable of demonstrating causality; hence, we conducted a subgroup analysis based on study type (cohort study or case–control study). Moreover, a subgroup analysis was carried out according to the quality of included studies (high-quality, not high-quality). Sensitivity analysis was conducted to assess the effect of a single study on the pooled results by reducing each study one time (36). All analysis was conducted by using Stata version 15.0 (Stata Corporation, College Station, TX, USA).

## Results

**Figure 1:**
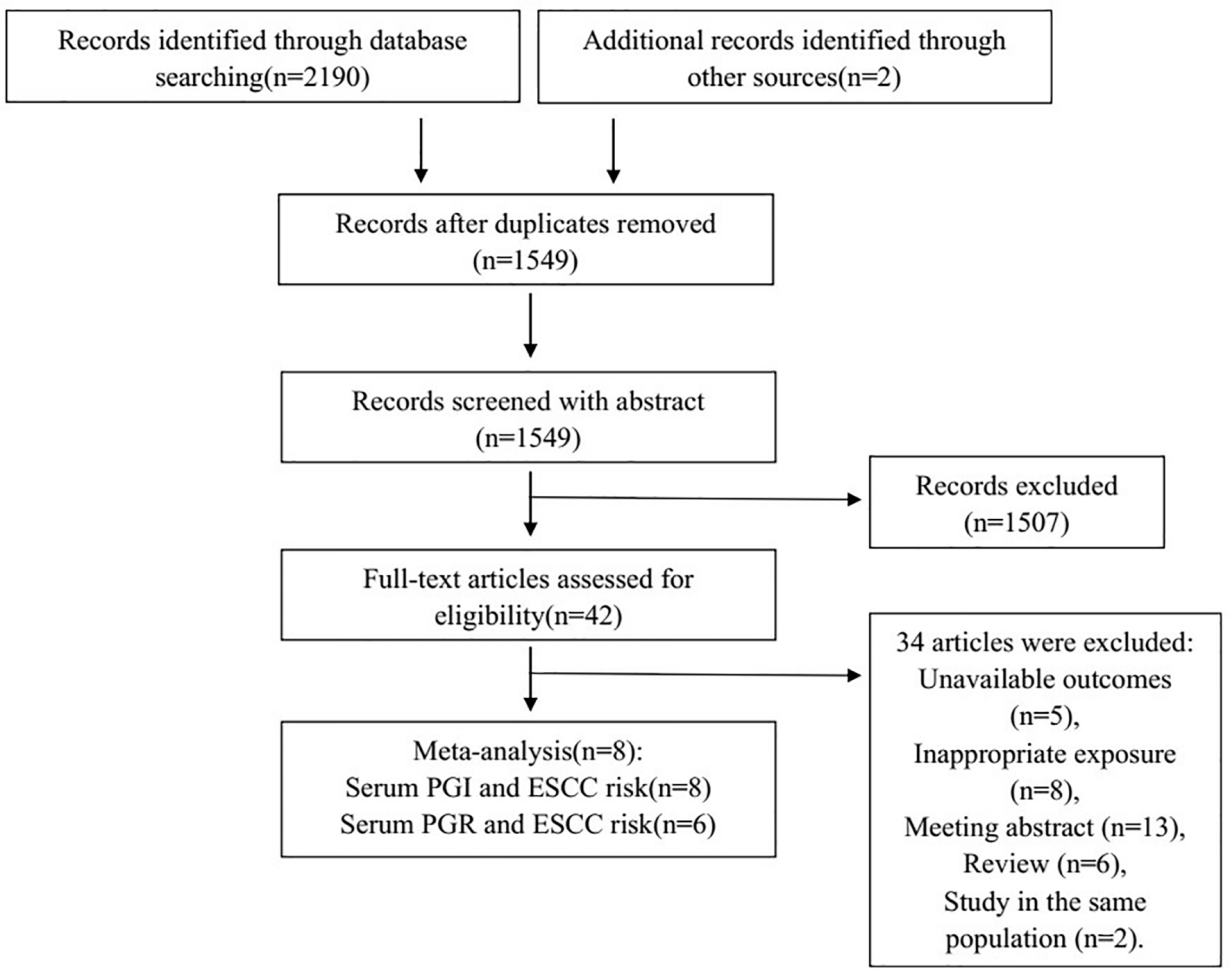
Flowchart of selection of eligible studies. PGI, pepsinogen I; PGR, pepsinogen I: pepsinogen II ratio; ESCC, esophageal squamous cell carcinoma.

A total of 2,190 studies were found from three databases (548 from PubMed, 485 from Embase, and 1,157 from Web of Science), and two studies were found from reference tracking. A total of 1,549 studies were left after duplicate removal. Of 42 studies for further full-text reviewing after the abstract screening, 34 studies were excluded for the reason of unavailable outcomes of interest, inappropriate exposure, conference abstract, review, and studies in the same population. Finally, eight studies (17–24) were included in the meta-analysis. **Figure 1** shows the flowchart of literature screening.

**Table 1** shows a summary of the characteristics of the included studies. The mean or median of age ranged from 40 to 85 years. Three studies (21–23) were carried out in China, one in Sweden (17), one in Japan (18), one in Iran (19), one in Finland (20), and one in Germany (24). Five were case–control studies (17–19, 21, 24), and three were prospective studies [one nested case–control study (20), one case–cohort study (22), and one cohort study (23)]. Six studies (17–22) adjusted for smoking and alcohol drinking when estimating the effect, and the other two studies did not (23, 24). For the study quality, six studies (17–22) were assessed as having good quality, and the other two were assessed as having fair quality (23, 24) (**Supplementary Tables S2, S3**).

**Table 1:** Characteristics of the included studies.

First author, Publication year, Country	Study design	Follow-up years (mean or maximum), or study period	Number cases/controls for case–control study, or number of total subjects for the cohort study	Age, years	Sex, males%	Biomarkers, boundary point, OR (95% CI) or HR (95% CI)	Adjustment
Ye, 2004, Sweden (17)	Case–control	1995–1997^c^	85/499^d^	Mean Case: 64 Controls: 69	Cases: 69% Controls: 83%	PGI PGI<28 μg/l vs. PGI ≥28 μg/l: 4.3 (1.9–9.6)^f^	Age, sex, education, consumption of fruits and vegetables, body mass index, tobacco smoking, alcohol consumption
Iijima, 2009, Japan (18)	Case–control	2004–2008^c^	100/100^d^	Mean (SD) Cases: 68.8 (7.1) Controls: 68.4 (6.7)	Cases: 90% Controls: 90%	PGI PGI<25 μg/l vs. PGI ≥25 μg/l: 3.1 (1.5–6.2)^f^ PGR PGI/PGII ratio ≤2 vs. PGI/PGII ratio>2: 3.3 (1.6–7.0) ^f^	BMI, smoking, drinking, *H. pylori* infection
Ren, 2009, China (22)	Case–cohort	5.25^a^	29,584^e^	Median (IQR) Case: 52 (44–59) Control: 56 (48–61)	45.5%	PGI PGI ≤50 μg/l vs. PGI >50 μg/l: 1.64 (0.89–3.00)^g^ PGR PGI/PGII ratio ≤3 vs. PGI/PGII ratio >3: 1.10 (0.59–2.04)^g^	Age, sex, history of smoking, alcohol consumption body mass index, *H. pylori* seropositivity
Cook, 2010, Finland (20)	Nested case–control	1985–2005^c^	79/94^d^	Mean (SD) Cases: 57.7 (4.6) Controls: 58.1 (4.8)	100%	PGI PGI ≤50 μg/l vs. PGI >50 μg/l: 3.68 (1.51–8.97)^f^ PGR PGI/PGII ratio ≤3 vs. PGI/PGII ratio >3: 4.32 (1.74–10.71)^f^	Age, date of blood draw, education, duration and intensity of cigarette smoking, alcohol, body mass index (BMI), fruit consumption, and vegetable consumption
Venerito, 2011, Germany (24)	Matched case–control	2006–2010^c^	75/75^d^	Cases Mean (SD): 64.9 (9.0) Controls: 63.2 (8.1)	Cases: 69.3% Controls:69.3%	PGI/PGII ratio ≤3 or PGI ≤70 μg/l vs. PGI/PGII ratio >3 or PGI >70 μg/l: 1.17 (0.54–2.56)^f^	Sex-matched and age-matched
Nasrollahzadeh, 2012, Iran (19)	Case–control	2003–2007^c^	293/524^d^	Mean (SD) Cases: 64.4 (11.1) Controls: 65.5 (10.4)	Cases: 50.2% Controls: 48.8%	PGI PGI ≤55 μg/l vs. PGI>55 μg/l: 1.39 (0.93–2.09)^f^ PGR PGI/PGII ratio ≤3 vs. PGI/PGII ratio>3 1.50 (0.85–2.60)^f^	Ethnicity, alcohol consumption, tobacco or opium use, education level, vegetable/fruit consumption
Xue, 2013, China (23)	Cohort	15^b^	1501^e^	Mean (SD) 45.3 (12.2)	36.9%	PGI PGI ≤70 μg/l vs. PGI >70 μg/l: 2.55 (0.51–12.78)^f^ PGR PGI/PGII ratio ≤3 vs. PGI/PGII ratio >3: 0.32 (0.04–2.61)^f^	–
Ekheden, 2020, China (21)	Case–control	2010–2014^c^	1210/1978^d^	40–85	Cases: 68% Controls: 69%	PGI PGI ≤55 μg/l vs. PGI>55 μg/l: 1.61 (1.33–1.96)^f^	Age, sex, education, marital status, occupation, family wealth score, BMI 10 year before, tea drinking, history of esophageal cancer, smoking status, alcohol consumption, H. pylori serostatus, filled teeth and frequency of daily toothbrushing

PGI, pepsinogen I; PGR, pepsinogen I: pepsinogen II ratio; OR, odds ratio; HR, hazard ratio; CI, confidence interval.

a Mean of follow-up years.

b Maximum of follow-up years.

c Study period.

d Number cases/controls for the case–control study.

e Number of total subjects for the cohort study.

f OR (95% CI).

g HR (95% CI).

**Figure 2:**
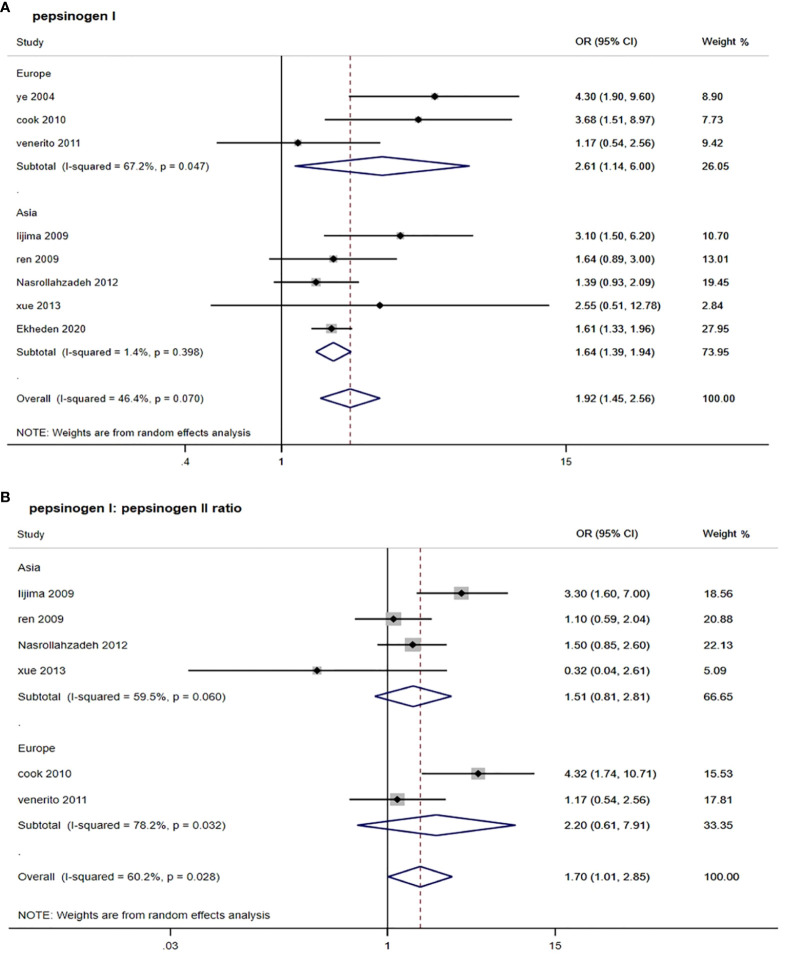
Forest plots from random-effect meta-analysis of the association between PGI and PGR and risk of esophageal squamous cell carcinoma. **(A)** Pepsinogen I; **(B)** pepsinogen I: pepsinogen II ratio. PGI, pepsinogen I; PGR, pepsinogen I: pepsinogen II ratio; OR, odds ratio; CI, confidence interval.

**Figure 3:**
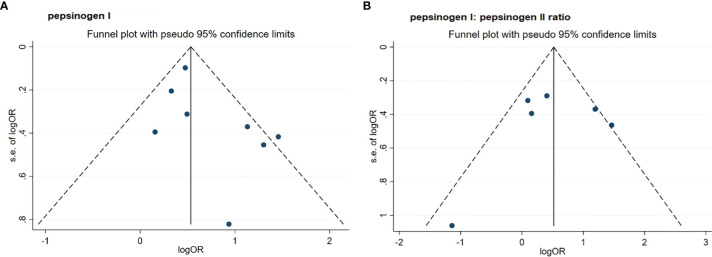
Funnel plots of standard error by log odds ratio for the association of PGI **(A)** and PGR **(B)** with risk of esophageal squamous cell carcinoma. **(A)** pepsinogen I; **(B)** pepsinogen I: pepsinogen II ratio. PGI, pepsinogen I; PGR, pepsinogen I: pepsinogen II ratio; OR, odds ratio.

As shown in **Table 2** and **Figure 2**, by using the random-effect model and comparing the low with high level, the pooled result showed that a low serum PGI level was associated with an increased ESCC risk (OR: 1.92, 95% CI: 1.45–2.56) with a moderate heterogeneity (*I* ^2^ = 46.4%, *P*_-heterogeneity_ = 0.070). The Egger’s test (*P* = 0.156) and the funnel plot (**Figure 3**) did not show any publication bias. Sensitivity analyses by omitting each study a time showed that none of these studies had a substantial influence on the overall results (**Supplementary Figure S1**), indicating that the results are robust. The stratified analysis by region yielded similar results in both Asia (OR: 1.64, 95% CI: 1.39–1.94, *I* ^2^ = 1.4%, *P*_-heterogeneity_ = 0.398) and Europe (OR: 2.61, 95% CI: 1.14–6.00, *I* ^2^ = 67.2%, *P*_-heterogeneity_ = 0.047) (**Table 2**). However, when stratified by study quality, a significant difference was observed when pooling studies with high quality (OR: 2.05, 95% CI: 1.48–2.84, *I* ^2^ = 57.9%, *P*_-heterogeneity_ = 0.036) rather than with low quality (OR: 1.36, 95% CI: 0.67–2.73, *I* ^2^ = 0.0%, *P*_-heterogeneity_ = 0.393).

**Table 2:** Meta-analysis on association of esophageal squamous cell carcinoma risk with PGI and PGR.

	Number of studies	PooledOR (95% CI)^a^	*I* ^2^ (%)	*P*_-heterogeneity_ ^b^
**PGI**				
All studies	8	1.92 (1.45–2.56)	46.4%	0.070
Regions				
Asia	5	1.64 (1.39–1.94)	1.4%	0.398
Europe	3	2.61 (1.14–6.00)	67.2%	0.047
Study type				
Cohort study	3	2.20 (1.31–3.70)	9.3%	0.332
Case–control study	5	1.86 (1.30–2.64)	59.5%	0.042
Study quality				
High	6	2.05 (1.48–2.84)	57.9%	0.036
Low	2	1.36 (0.67–2.73)	0.0%	0.393
**PGR**				
All studies	6	1.70 (1.01–2.85)	60.2%	0.028
Regions				
Asia	4	1.51 (0.81–2.81)	59.5%	0.060
Europe	2	2.20 (0.61–7.91)	78.2%	0.032
Study type				
Cohort study	3	1.44 (0.42–4.89)	75.8%	0.016
Case–control study	3	1.78 (1.01–3.15)	51.9%	0.125
Study quality				
High	4	2.07 (1.14–3.75)	66.5%	0.030
Low	2	0.89 (0.32–2.51)	23.0%	0.254

PGI, pepsinogen I; PGR, pepsinogen I: pepsinogen II ratio.

a A random-effect model was adopted.

b p value from Q-test.

For six studies that assessed the association between serum PGR and risk of ESCC, the pooled OR (95% CI) was 1.70 (1.01, 2.85) when comparing the low with high level (**Figure 2** and **Table 2**). The *I* ^2^ statistic was 60.2% and the *P* value for Q-test was 0.028, suggesting a marginal heterogeneity. The Egger’s test (*P* = 0.812) and the funnel plot (**Figure 3**) did not display any obvious publication bias. Sensitivity analyses by omitting each study at a time showed that none of these studies had a substantial effect on the overall results (**Supplementary Figure S1**), indicating that the results are stable. In stratified analysis by region, the association of serum PGR and ESCC risk was observed in neither group (**Table 2**) when comparing the low and high serum PGR levels. In stratified analysis by study quality and study type, a significant association was only observed when four studies with high quality were pooled (OR: 2.07, 95% CI: 1.14–3.75, *I* ^2^ = 66.5%, *P*_-heterogeneity_ = 0.030) or three studies with case–control design were pooled (OR:1.78, 95% CI: 1.01–3.15, *I* ^2^ = 51.9%, *P*_-heterogeneity_ = 0.125).

## Discussion

In the present study, we found that both low level of serum PGI and low level of serum PGR were associated with an increased risk of ESCC.

Smoking and drinking were two risk factors for ESCC; hence, we conducted a subgroup analysis based on whether these two risk factors were adjusted. Two studies [one cohort study (24) and one matched case–control study (23)] did not adjust for smoking and alcohol drinking, and these two studies (23, 24) were also deemed to have low quality (23, 24); hence, the stratified analysis according to whether to adjust for smoking and alcohol drinking was the same with the stratified analysis by the study quality. After excluding these two studies (23, 24), similar risk effects to the main analysis were observed for both PGI and PGR when only the studies with high quality were considered. This finding suggests that although there may be complex interrelationships between PGI, PGR, and ESCC, the studies should be conducted with high quality and most confounders should be considered in future studies.

For PGI, the subgroup analysis by region found that the association was stronger in European than in Asian, suggesting the geographical or ethnic variation. This was consistent with the finding by Islami et al. (25). When stratified by study type, a similar risk effect was observed when cohort studies and case–control studies were pooled separately, further indicating that the results were stable. For PGR, a significantly positive association of serum PGR with ESCC risk was observed when three case–control studies were pooled. However, no significant association was observed when longitudinal studies, studies in Asia, or studies in Europe were pooled; this might be due to the high heterogeneity among studies in these subgroups. De Vries et al. (11) proposed that the association between gastric atrophy determined by PGR and ESCC can be explained by confounding factors, such as smoking, after proving the connection between gastric atrophy and small cell lung carcinoma. However, the present systematic review found a statistically significant association between PGR and ESCC risk after adjusting for smoking and drinking status. Kamangar et al. (28) showed that lower serum PGR was associated with a continuous increase in the risk of esophageal squamous dysplasia, the precursor lesion of ESCC, which was consistent with results in our meta-analysis.

Gastric atrophy has been associated with ESCC, but its causal relevance has been questioned. It was suggested that gastric atrophy might be related to ESCC because of bacterial overgrowth and N-nitrosation reactions caused by the reduced gastric acid production during gastric atrophy or duodenal reflux (37–39). Some studies (40, 41) have found that *Helicobacter pylori* infection was associated with a higher risk of ESCC, and gastric atrophy may be an intermediate step in the pathway from *Helicobacter pylori* infection to ESCC. However, since most studies did not provide information on the combined stratification of *Helicobacter pylori* and serum PGI or PGR, we cannot examine it directly in the present meta-analysis.

There are some advantages in this study. Firstly, as many eligible studies as possible were included in this study. Secondly, several subgroup analyses were conducted to identify potential sources of heterogeneity. Several limitations also existed. Firstly, substantial heterogeneity was observed among included studies. This heterogeneity may be due to various factors, such as diversity in the population characteristics, serum pepsinogen detection methods, and study design. Secondly, there is no consensus on the best cutoff point for serum pepsinogen biomarkers. Dichotomous comparisons may not be the most efficient use of data in estimating the association between gastric atrophy and ESCC risk. These comparisons may be misleading, because any cutoff point will put people with considerably different risks into the same category. Therefore, it is urgent to conduct the delicate well-designed longitude studies and dose–response analyses.

## Conclusion

In summary, this systematic review with meta-analysis, although based on a limited number of studies, suggested that both low level of serum PGI and low level of serum PGR were related to an increased risk of ESCC, respectively. This present study provides evidence for using serum pepsinogen biomarkers in predicting ESCC. More delicate well-designed cohort studies with high study quality are needed and dose–response analyses should be performed.

## Data Availability Statement

Publicly available datasets were analyzed in this study. The data are available by contacting the corresponding author or extracting from originally published research.

## Author Contributions

XL and WZ conceived and designed this study. Z-XY and L-BY researched the literature. Z-XY and PH performed the statistical analysis. Z-XY and PX drafted the manuscript. XL, XX, YL, WZ, and L-BY edited and reviewed the manuscript. All authors contributed to the article and approved the submitted version.

## Funding

This study was supported by the National Key R&D Program of China (No. 2018YFE0208000), the Guangdong Basic and Applied Basic Research Foundation (No. 2019A1515011599), and the Science and Technology Program of Guangzhou City (No. 202102080404). The funders had no role in the design, analysis, or writing of this manuscript.

## Conflict of Interest

The authors declare that the research was conducted in the absence of any commercial or financial relationships that could be construed as a potential conflict of interest.

## Publisher’s Note

All claims expressed in this article are solely those of the authors and do not necessarily represent those of their affiliated organizations, or those of the publisher, the editors and the reviewers. Any product that may be evaluated in this article, or claim that may be made by its manufacturer, is not guaranteed or endorsed by the publisher.
