# Supplementary material for: Association of Serum Pepsinogens With Esophageal Squamous Cell Carcinoma Risk: A Systematic Review and Meta-Analysis
Source: Front Oncol. 2022 Jun 30;12:928672. doi: 10.3389/fonc.2022.928672 (PMC9280489; doi:10.3389/fonc.2022.928672)
Supplement: Supplementary file 1 [file DataSheet_1.docx]

**Supplementary Material**

Supplementary Table S1. Literature search strategy

Supplementary Table S2. Quality assessment using the Newcastle–Ottawa scale for cohort studies

Supplementary Table S3. Quality assessment using the Newcastle–Ottawa scale for case-control studies and nested case-control studies

Supplementary Figure S1. Sensitivity analysis of the association of PGI (A) and PGR (B) and risk of esophageal squamous cell carcinoma

Abbreviation: PGI, Pepsinogen I; PGR, pepsinogen I: pepsinogen II ratio

Supplementary Table S1. Literature search strategy

| **PubMed** |
| --- |
| 1.(“PGI” OR “PGⅡ” OR “PGI:Ⅱ” OR “PGI/Ⅱ” OR “PGR” OR “PG” OR “pepsinogen” OR “gastric atrophy” OR “pepsinogens”[Mesh] OR “gastritis, atrophic”[Mesh]) |
| 2. (esophag* OR oesophag*) AND (cancer OR carcinoma OR tumor OR neoplasms) |
| 3. “esophageal squamous dysplasia” OR “esophageal squamous cell carcinoma” OR “esophageal squamous cell carcinoma”[Mesh] OR “esophageal neoplasms”[Mesh] |
| 4. 2 OR 3 |
| 5. 1 AND 4 |
| **Embase** |
| 1.‘PGI’:ti,ab,kw OR ‘PGⅡ’:ti,ab,kw OR ‘PGI:Ⅱ’:ti,ab,kw OR ‘PGI/Ⅱ’:ti,ab,kw OR ‘PGR’:ti,ab,kw OR ‘PG’:ti,ab,kw OR ‘pepsinogen’:ti,ab,kw OR ‘gastric atrophy’:ti,ab,kw OR ‘atrophic gastritis’:ti,ab,kw OR ‘pepsinogen’/exp OR ‘atrophic gastritis’/exp OR ‘gastric atrophy’/exp |
| 2.‘esophageal squamous dysplasia’:ti,ab,kw OR ‘esophageal squamous cell carcinoma’:ti,ab,kw OR ‘esophageal squamous cell carcinoma’/exp OR ‘esophagus tumor’/exp |
| 3. 1 AND 2 |
| **Web of Science** |
| 1. TS=((esophag* OR oesophag*) AND (cancer OR carcinoma OR tumor OR neoplasms)) |
| 2. TS=(“esophageal squamous dysplasia” OR “esophageal squamous cell carcinoma”) |
| 3. 1 OR 2 |
| 4. TS=(“PGI” OR “PGⅡ” OR “PGI:Ⅱ” OR “PGI/Ⅱ” OR “PGR” OR “PG” OR pepsinogen OR “gastric atrophy”) |
| 5. 3 AND 4 |

Supplementary Table S2. Quality assessment using the Newcastle–Ottawa scale for cohort studies

|  | Selection | | | | | Comparability | Outcome | | | total | | grade |  |
| --- | --- | --- | --- | --- | --- | --- | --- | --- | --- | --- | --- | --- | --- |
| Study | | Representa-  tiveness  of exposed | Selection of non-exposed | Exposure ascertainment | Outcome not present  at start | Adjustment  /matching | Outcome ascertainment | Follow-up duration | Lost to follow-up | |  |  | |
| Ren2009 | | 1 | 1 | 1 | 1 | 2 | 1 | 1 | 1 | | 9 | Good | |
| Xue2013 | | 1 | 1 | 1 | 1 | 0 | 0 | 1 | 1 | | 6 | fair | |

Supplementary Table S3. Quality assessment using the Newcastle–Ottawa scale for case-control studies and nested case-control studies

|  | Selection |  |  |  | Comparability | Outcome |  |  | Total | Grade |
| --- | --- | --- | --- | --- | --- | --- | --- | --- | --- | --- |
| Study | Case definition | Representativeness of cases | Control selection | Control definition | Adjustment/ matching | Exposure ascertainment | Same method | Response Rate |  |  |
| Case-control studies |  |  |  |  |  |  |  |  |  |  |
| Ye 2004 | 1 | 1 | 1 | 1 | 2 | 1 | 1 | 1 | 9 | good |
| Iijima 2009 | 1 | 0 | 0 | 1 | 2 | 1 | 1 | 1 | 7 | good |
| Venerito 2011 | 1 | 0 | 0 | 1 | 1 | 1 | 1 | 1 | 6 | fair |
| Nasrollahzadeh2012 | 1 | 1 | 1 | 1 | 2 | 1 | 1 | 1 | 9 | good |
| Ekheden 2020 | 1 | 1 | 1 | 1 | 2 | 1 | 1 | 0 | 8 | good |
| Nested case-control studies |  |  |  |  |  |  |  |  |  |  |
| Cook 2010 | 1 | 1 | 1 | 1 | 2 | 1 | 1 | 1 | 9 | good |


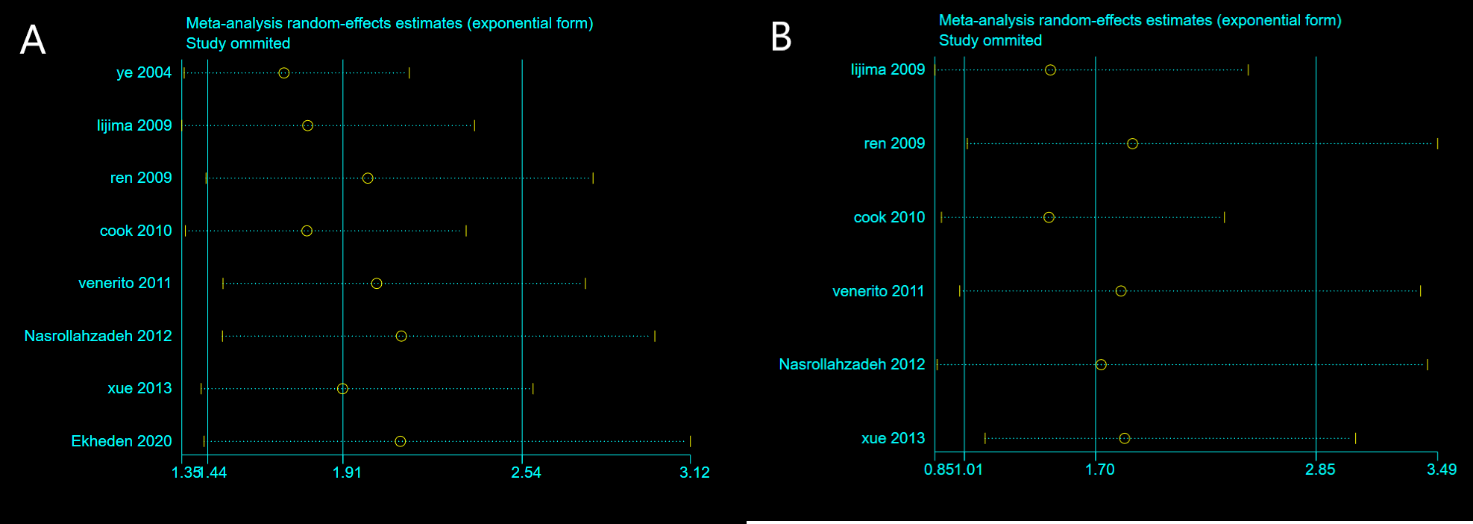


Supplementary Figure S1. Sensitivity analysis of the association of PGI (A) and PGR (B) and risk of esophageal squamous cell carcinoma.

Abbreviation PGI: Pepsinogen I; PGR: pepsinogen I: pepsinogen II ratio.
